# “Traditional” and “Healthy” Dietary Patterns Are Associated with Low Cardiometabolic Risk in Brazilian Subjects

**DOI:** 10.1155/2018/4585412

**Published:** 2018-11-19

**Authors:** Brenda Kelly Souza Silveira, Juliana Farias de Novaes, Nínive de Almeida Reis, Larissa Pereira Lourenço, Ana Helena Moretto Capobiango, Sarah Aparecida Vieira, Helen Hermana Miranda Hermsdorff

**Affiliations:** ^1^MSc, Department of Nutrition and Health, Universidade Federal de Viçosa, Viçosa, Minas Gerais, Brazil; ^2^PhD, Department of Nutrition and Health, Universidade Federal de Viçosa, Viçosa, Minas Gerais, Brazil; ^3^Undergraduate Student, Department of Nutrition and Health, Universidade Federal de Viçosa, Viçosa, Minas Gerais, Brazil; ^4^PhD, Department of Integrated Education in Health, Universidade Federal do Espírito Santo, Espírito Santo, Brazil

## Abstract

This study aimed at determining the dietary patterns and investigating their association with cardiometabolic risk markers in a brazilian population at risk. This transversal study was carried out with data of 265 patients (*n* = 123 M/172 W, age 42 ± 16 years) of the Cardiovascular Health Care Program—PROCARDIO-UFV, Brazil—who had their first appointment between 2012 and 2017. A 24-hour recall was applied. The dietary patterns were determined by Principal Component Analysis. Anthropometric, clinical-metabolic, sociodemographic, and lifestyle data were collected through medical record analysis. Five patterns were identified: “Traditional”, “Caloric”, “Unhealthy”, “Healthy,” and “Healthy Snacks”. In bivariate analysis, the “Healthy” pattern was negatively associated with WC (waist circunference), BMI (body mass index), WHR (waist-to-hip ratio), SBP (systolic blood pressure), fasting glucose, TG/HDL, LDL/HDL, and TG/HDL values and positively to HDL. The “Traditional” pattern was positively associated with adiposity indicators (WC, BMI, and WHR) and negatively associated with body fat, TyG (triglyceride-glucose index), HDL, and LDL (*P* < 0.05). However, in adjusted models of Poisson regression, individuals with positive factor score (higher adherence) in the “Traditional” and “Healthy” patterns had less occurrence of abdominal obesity (PR 0.85; 95% CI 0.74–0.99/PR 0.88; 95% CI 0.02–0.76), as well as dyslipidemia (PR 0.06; 95% CI 0.02–0.51/PR 0.03; 95% CI 0.01–0.27), diabetes (PR 0.05; 95% CI 0.01–0.45/PR 0.02; 95% CI 0.01–021), and hypertension (PR 0.06; 95% CI 0.02–0.50/PR 0.02; 95% CI 0.01–0.21). A greater adherence to the “Healthy” pattern was associated with lower values to cardiometabolic risk markers and less occurrence of chronic diseases, while the “Traditional” pattern presented contradictory results.

## 1. Introduction

Cardiovascular diseases (CVD) and their complications are the main causes of increased mortality, accounting for 31% of deaths [1, 2]. Although genetic factors may contribute to their development, lifestyle factors, such as sedentary lifestyle, alcohol intake, and unhealthy eating habits, are the main determinants for CVD development and progression [3]. Several researchers have investigated nutrients and bioactive compounds capable of modulating inflammation, oxidative stress, and other mechanisms responsible for CVD development [4–6]. However, in recent decades, the scientific literature has also analyzed dietary patterns and not just the effect of a single nutrient on human health [7–9], because they are ingested simultaneously, acting synergistically in the body through complementary or antagonistic mechanisms. In this sense, the cluster analysis techniques and the principal component analysis or PCA (factorial) are the statistical methods more frequently used when determining dietary patterns [10, 11]. PCA has been frequently used in nutritional epidemiology confirming or identifying a new pattern by assembling food groups with few components and minimal loss of the original information from the Food Survey [12]. In turn, dietary patterns with high industrialized food which are predominantly high in sodium, trans fats, and sugar have been designated to increase the risk of chronic diseases development and death [13, 14], while patterns with predominant *in natura* foods are associated to better health [15, 16]. Although several studies have described the dietary patterns in different populations [9, 14, 17], few have investigated their association with multiple risk factors for CVD. To our knowledge, no Brazilian study has investigated the association between dietary patterns and cardiometabolic risk markers in population groups at risk for CVD. Therefore, the objective of this study is to determine the dietary patterns in a population receiving nutritional accompaniment because at least one risk factor for CVD is present and investigate the potential association of these patterns with cardiometabolic risk markers.

## 2. Methods

### 2.1. Subjects

This transversal study was carried out with 295 individuals (172 women and 123 men), adults and elders (42 ± 16 years), and assisted by the Cardiovascular Health Care program (PROCARDIO-UFV) of the Universidade Federal de Viçosa—UFV (Brazil). This program performs continuous nutritional intervention in the university community and is registered in the Brazilian Registry of Clinical Trials ReBEC identifier number RBR-5N4Y2G. The program's inclusion criteria are as follows: patients of both genders, who had their first appointment between March 2012 and July 2017, age ≥20 years, being an UFV employee/employee's spouse or offspring or student, having been diagnosed with cardiovascular disease or occurrence of cardiometabolic risk factor, such as overweight (body mass index ≥25 kg/m^2^), hypertriglyceridemia (≥150 mg/dL), and hypercholesterolemia (≥200 mg/dL), low HDL (men <40 mg/dL and women <50 mg/dL), blood pressure ≥130/≥ 85 mmHg or systemic arterial hypertension diagnosis (systolic blood pressure ≥140 and/or diastolic blood pressure ≥90 mmHg), fasting blood glucose ≥100 mg/dL or diagnosis of diabetes mellitus (fasting blood glucose ≥126 mg/dL), and/or medical referral. The programme methodology has already been previously described [18, 19]. We excluded five patients who did not complete the interview and six others who underestimated or overestimated food consumption as described in the next section. Our study was approved by the Ethics Committee on research with human beings from UFV (of. Ref. no. 066/2012/CEPH), according to the resolution 466/2012 of the National Health Council. All participants in this study read and signed the term of free and informed consent in accordance to the principles of the Helsinki declaration.

### 2.2. Food Consumption

Patients underwent a 24-hour recall (24HR), which, according to Willett 1998 [20], may be sufficient to estimate food and nutrients intake in a population, provided that the sample has sufficient size. To guarantee data collection quality, we adopted the “multiple-pass” technique [21] and used photographic albums of presets and standard utensils for measures performed at home. All interviewers (nutritionists and undergraduation students of nutrition) were trained during four months and supervised during the first interviews.

We excluded five patients who did not complete the interview and six others who underestimated (<500 kcal/day) or overestimated (>4000 kcal/day) food consumption [20]. For PCA, food recorded in milliliters/day (mL/d) was converted to grams/day (g/d) according to the Density Database Table, Version 2.0 [22]. A total of 217 different foods were reported in 24HR and were collapsed into 20 food groups according to chemical similarity, beginning with those consumed by less than 5% of the sample [11, 23].

### 2.3. Anthropometry

Weight, height, and waist circumference (WC) were measured according to the protocol standardized by PROCARDIO-UFV, previously described [24]. The body weight was measured in an electronic digital scale (Toledo 2098PP, São Bernardo do Campo, Brazil) with a maximum capacity of 200 kg and a precision of 50 g. The height was determined in a stadiometer (Stanley, CMS, England), with a maximum extension of 2 m and precision of 0.5 mm. The WC was measured on top of the umbilical scar. The body mass index (BMI) was calculated and classified. Overweight and obesity were considered at BMI ≥25.0 kg/m^2^ and BMI ≥ 30.0 kg/m^2^ [25] for adults and BMI ≥28.0 kg/m^2^ and ≥30.0 kg/m^2^ for elders, respectively [26]. Abdominal obesity was accounted for when WC ≥ 90 and ≥80 cm for men and women, respectively [27].

Waist-to-height ratio (WHtR) and waist-to-hip ratio (WHR) were calculated, and an increased cardiometabolic risk was considered when WHtR >0.5 [28] and WHR >1.0 for men and >0.85 for women [25].

Body fat (BF) was estimated through horizontal tetrapolar electric bioimpedance (Biodynamics® 310 model, Washington, USA), according to the protocol proposed by Lukaski et al. [29], The cut-off points for BF excess values were >20% for men and >30% for women [30].

### 2.4. Cardiometabolic Risk Markers

A qualified professional collected blood after 12 hours with disposable material and venipuncture. The enzymatic colorimetric method was used to analyze serum concentrations of glucose, HDL and LDL cholesterol, triglycerides (TG), and uric acid, while the ultrasensitive immunoturbidimetry method assessed serum concentration of ultrasensitive C-reactive protein (CRP). The CT/HDL, TG/HDL, and LDL/HDL ratios were calculated as well as the triglyceride-glucose index (TyG) which was calculated according to the formula Ln (TG (mg/dL) × fasting blood glucose (mg/dL)/2) [31]. Increased values were defined as fasting glucose ≥100 mg/dL, uric acid ≥6 mg/dL, CRP ≥ 3 mg/dL [32], and LDL/HDL ratio ≥3.3 [33].

Blood pressure was measured using a mechanical mercury sphygmomanometer (Missouri®, São Paulo, Brazil) with approximately 02 mmHg, according to the technique described in the VI Brazilian Hypertension Guidelines [34]. In addition, the participants were the ones to report medical diagnosis of diabetes, hypertension, and dyslipidemias and the use of medications.

### 2.5. Sociodemographic and Lifestyle Data

During an interview, participants reported age, sex, schooling, income (in minimum wages), marital status (single, married, stable union, divorced, or widowed), the type of link with UFV (employee, student or relative), smoking habit (smokers, ex-smokers or nonsmokers), alcoholism (do not drink, drink sometimes, drink daily, or ex-alcoholic), and regular practice of physical activity (>150 min/week) (yes or no).

### 2.6. Statistical Analysis

Exploratory factor analysis was performed using the principal component analysis (PCA). The Kaiser–Meyer–Olkin measurement of sampling adequacy (KMO) and the Bartlett test of sphericity (BTS) were estimated and considered appropriate if > 0.6 and <0.05, respectively [35, 36]. The communalities (*h*^2^) were calculated and an anti-image model was inspected to verify the adequacy of each variable to the PCA test, where the KMO value was presented in the diagonal of this matrix, being higher than 0.5 for all variables [37]. The orthogonal varimax rotation was performed to make the values interpretable.

Factor retention was based on the Kaiser criteria (eigenvalue >1.0) and the inflection point of the eigenvalues from the Cattell scree test (screeplot) [38], suggesting the retention of 10 and 7 factors, respectively. For the final decision, we considered the formation of interpretable patterns, and we chose the criterion of the Cattel chart with the exclusion of two factors [39, 40]. Food groups with factor score >0.25 were considered as nonsignificant in the pattern [41]. When a food group saturated with positive score >0.25 in two patterns, the one with the highest score prevailed. When a food group saturated with opposing charges (positive and negative) in two patterns, it was maintained in both.

The patterns were named according to the food items included and the nomenclature adopted in other studies [8, 23] to facilitate data comparison. Finally, the factor scores for each dietary pattern were calculated for each participant. A positive factor score indicates a high intake of foods within the respective pattern, while a negative factor score indicates a low intake.

The characterization variables of the sample were described by means of frequency distribution measures. The normality of the data was evaluated by the Kolmogorov–Smirnov test. Student's *t* test was used to compare the mean scores in the dietary patterns according to self-reported diseases. In the bivariate analysis, the regression coefficient and the confidence interval were estimated through simple linear regression for analyzing potential association of cardiometabolic risk factors (dependent variables) and dietary patterns (independent variables). These analyses were performed in the Statistical Package for Social Science (SPSS® 24.0, Chicago, IL, USA, 2016). Poisson regression models were used to evaluate the association between cardiometabolic risk factors (dependent variables) and positive factor score in dietary patterns (independent variable). This analysis was performed in STATA software, version 13.0. A significance level of 5% was considered for all tests.

Statistical power was calculated in the OpenEpi software online version 3.01 [42], with a 95% confidence interval. Two groups were considered for this calculation: exposed (positive score in the “Traditional” pattern) and not exposed (positive score in the “Healthy” pattern) and the prevalence of overweight, dyslipidemia, hypertension, and diabetes. The power of the tests was, on average, 87.1%.

## 3. Results

This study included 265 subjects with cardiometabolic risk. The sociodemographic and clinical characteristics of the sample are presented in Table 1. A considerable prevalence of chronic diseases is observed as expected in the study sample.

Regarding PCA, the sample was adequate according to the KMO and BTS tests (KMO = 0.64 and BTS <0.001). The food groups used for analysis are described in Table 2.

Five dietary patterns were identified from the PCA test, which explained 39.7% of the dietary intake variance. The “Traditional” pattern, composed of rice and tubers, beans, vegetable oils, nonleafy vegetables, meats, fish, and eggs (grilled, cooked or roasted), explained 10.9% of the data variance. The second pattern named “Caloric” was composed of meat, offal and eggs (fried), processed meat, sweets and sugar, and soft beverages and artificial juices, explained 8.2% of the variance. The “Pastry” pattern was represented by fast food and pasta, with negative saturation for milk (whole or skimmed), sweets, and sugar accounting for 7.0% of the variance. In the “Healthy” pattern, whole grain food and nuts, milk, dairy, fruits, and natural juices were main groups. In addition, margarine/butter, sauces, and mayonnaise, as well as alcoholic beverages, coffee, and tea saturated with negative score, i.e., were inversely associated. This pattern explained 6.9% of the variance. The last pattern named “Healthy Snacks” was represented by leafy vegetables, chicken salad sandwich and presented negative saturation for fast food and pasta, explaining 6.7% of the dietary intake variance (Table 3).

**Figure 1:**
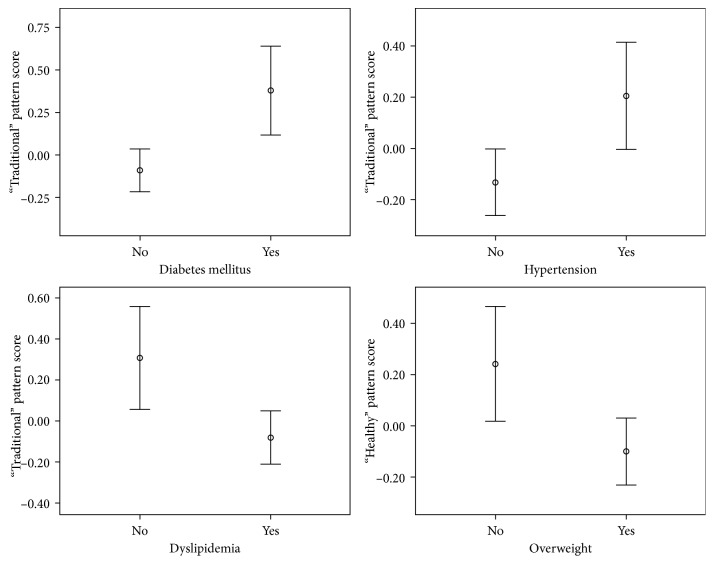
Scores of the “Traditional” and “Healthy” dietary patterns (mean and 95% confidence interval), according to self-report of chronic diseases, Brazil, 2017. Student's *t* test (*P* < 0.05 for all variables).

The mean factor score of the “Healthy” pattern was higher among subjects with normal weight than those who were overweight (Figure 1).

Moreover, in the bivariate linear regression, the factorial score of the “Traditional” pattern was positively associated with WC, BMI, WHR, SBP (systolic blood pressure), and fasting glucose values and negatively associated with BF%, TyG, HDL, and LDL. The factor score of the “Healthy” pattern was negatively associated with WC, BMI, WHR, SBP, fasting glucose, CT/HDL, LDL/HDL, and TG/HDL ratios and positively associated with HDL (Table 4).

In the prevalence analysis, subjects with a positive factor score (greater adherence) in the “Traditional” and “Healthy” patterns had a lower occurrence of abdominal obesity, dyslipidemia, diabetes mellitus, and hypertension increased, increased WHR and WHtR (*P* < 0.05). Those with a positive factor score in the “Traditional” pattern also had lower occurrence of overweight and an increased LDL/HDL ratio (Table 5).

## 4. Discussion

This cross-sectional study, conducted with adults presenting cardiometabolic risk, identified five eating patterns (“Traditional”, “Caloric”, “Healthy,” and “Healthy Snacks”). These patterns are similar to those described in previous publications that have also used PCA and commonly interpret a healthy pattern, an unhealthy pattern, and an intermediate pattern [7, 43, 44]. The “Traditional” pattern is frequently present in Brazilian studies conducted with different age groups [17,45–47]. This pattern consists of foods that characterize the Brazilian eating habits (rice and tubers, beans, meats, and vegetable oils) and has received several denominations, such as “Brazilian,” “Traditional,” or “Prudent” [44, 45].

In this study, diabetics and hypertensives presented a higher mean score in the “Traditional” pattern. In the bivariate analysis, this pattern was positively associated with indicators of adiposity, blood pressure, fasting glycemia, and negative percentage of body fat, TyG, HDL, and LDL. However, in a model adjusted for confounding factors, subjects with higher adherence in this pattern presented low overweight occurrence, increased LDL/HDL ratio, and diagnosis of dyslipidemia, diabetes, and hypertension. In the literature, the associations between the “Traditional” pattern and the cardiometabolic risk are controversial. In this sense, a study carried out with Brazilian adult women identified a similar pattern and considered the “Traditional” pattern a risk, denominating it as a “cost risk 1 dietary pattern”, because this pattern consists of low cost food [43]. In another Brazilian study, the “Traditional” pattern was positively associated with glycemia and BMI and negatively associated with TG and WHR [48]. Among Europeans, the “Traditional” pattern (potatoes, sautéed vegetables, oils and margarine, red and processed meat, coffee, and bread) was associated with a higher risk of CVD [7]. Other researchers have observed the protective effect of this pattern in several age groups [17, 23, 49], including less occurrence of obesity and risk behaviors for NCD. Foods in the “Traditional” pattern, such as vegetables, rice, beans, and eggs, are considered healthy, and the Food Guide for the Brazilian Population recommends them to be consumed daily in the context of a balanced and diversified diet [50]. However, some of the foods that make up this pattern, such as rice, tubers, and vegetable oils, are of higher caloric density. The negative relationship between the “Traditional” pattern and health status may be influenced by the excessive addition of oil and salt during the meal preparation, which can contribute to positive caloric balance, and, consequently, adiposity increase and dyslipidemias development [48]. In turn, a greater adherence to the “Healthy” pattern, characterized by a greater intake of whole grain foods, nuts, fruits and natural juices, milk and dairy products, and a low consumption of margarine, butter, oily sauces, alcohol, coffee, and tea, was associated negatively to the adiposity indicators, blood pressure, and fasting blood glucose. In a model adjusted for confounding factors, the “Healthy” pattern was associated to low occurrence of abdominal obesity, dyslipidemia, diabetes, and hypertension. This pattern resembles other cardioprotective patterns described in the literature and associated with low disease risk [15, 51, 52] and low mortality [53–55]. Therefore, the protective effect of this pattern is well established. In a cohort, the highest score in the “Prudent” pattern (wine, eggs, fruit, vegetables, fish, etc.), similar to our “Healthy” pattern, was associated with reduced CVD risk [7]. In a British elderly population, the “Mediterranean” pattern was associated with low mortality (reduction of 18% from highest to lowest tertile) [9]. Among elderly participants of the British Regional Heart Study, the second quartile of the “Prudent” pattern (fruits, vegetables, fish, legumes, rice, eggs, olive oil, etc.) was associated with low risk of CVD death [14]. The healthy patterns described in the literature are characterized by the ingestion of cardioprotective foods: vegetables, fruits, nuts, olive oil, and low ingestion of wine and saturated fats [54]. The ingestion of nuts and extra-virgin olive oil are related to a higher HDL serum concentration, a better control of lipemia, a lower risk of developing obesity, diabetes, and dyslipidemia [56, 57], and reduction of CVD deaths [57, 58]. Such benefits are attributed to the bioactive compounds present in these foods, such as polyphenols that have antioxidant effect and inhibit the oxidation of LDL. The main mechanism linked to CVD development is LDL oxidation caused by oxidative stress and associated with subclinical inflammation, endothelial dysfunction, and atherosclerosis [53, 59]. In this context, fruits and vegetables contribute to the reduction of these diseases, as they are sources of antioxidants and bioactive compounds that contribute to a better lipid profile and lower abdominal obesity [60, 61]. In addition to the intake of fruits, vegetables, olive oil, and nuts, cardioprotective diets are also characterized by the low intake of red meat and saturated fats, as these are associated with metabolic syndrome and risk factors for CVD [62, 63]. Nevertheless, white meat and fish have been related to protective food patterns [64]. The inverse association between the “Healthy” pattern with adiposity markers allows us to infer about the protective role of this pattern on CVD, considering that the increase of adiposity leads to a greater release of cytokines and inflammatory biomarkers that favor insulin resistance and atherosclerosis [65]. Moreover, this pattern resembles the standards considered effective in preventing and controlling CVD and its complications [66, 67].

The samples in our study were mainly of high level of education (60%) and mostly teachers and college students who spend a lot of time away from home, a factor that contributes to a greater consumption of fast food and processed foods [68, 69]. Although the increase in schooling is associated with increased income and access to food, food choices have multiple determinants [70, 71]. In this sense, a Brazilian study identified that subjects with a high level of education were divided into two groups: the one that had greater adherence to the “Healthy” pattern and the one that had greater adherence to the “High risk” pattern [43]. On the contrary, those with a low level of education had greater adherence to the “Pattern of risk and low cost”, similar to our “Traditional” pattern [43]. Therefore, increasing schooling contributes to access to food, but in itself does not ensure a better diet quality. Motivation, awareness, and other factors may be related. Finally, in relation to the method used for dietary pattern analysis, PCA has the advantage of being an empirical approach in the determination of dietary patterns, that is, no inferences are made about the composition of the patterns or their effects on health. Hence, this approach allows identifying specific characteristics of the alimentary habit of each population. However, food grouping and the choice of how many patterns will be retained occur subjectively, which may influence associations with assessed outcomes, as well as the constitution of patterns, and make it difficult to compare results. For this reason, this study was based on previous publications [8, 23] to carry out the grouping of foods. In view of the above, more studies are needed to investigate the relationship between the patterns and the cardiometabolic risk, in the context of the specificities of the food habit of each population.

## 5. Conclusion

A greater adherence to the “Healthy” pattern, similar to other cardioprotective patterns, was associated with the lower cardiometabolic risk outcome and less occurrence of chronic diseases, while the “Traditional” pattern presented contradictory results, being more studies needed to elucidate the relationship between “Traditional” Brazilian dietary pattern and the risk of chronic diseases, as well as the interference of sugars, oils, and salt in this relationship.

## Figures and Tables

**Table 1:** Sociodemographic, lifestyle, and clinical characteristics in cardiometabolic risk subjects (*n* = 295) in Brazil, 2017.

Variables	*n*	%
*Sex*		
*Female*	172	58.3
*Age (years)*		
<30	108	36.6
30–60	146	49.5
6–84	41	13.9
*Education*		
Primary or secondary (complete or incomplete)	127	39.6
College (complete or incomplete)	168	60.4
*Family Income*		
Until 4 minimum wages	209	67.4
>4 minimum wages	86	32.6
*Employment at UFV*		
Employee or relative	177	59.7
Student	118	40.3
*Smoking* ^*∗*^		
Smoker or ex-smoker	93	32.0
Never smoked	198	68.0
*Physical activity (>150 minutes per week)*		
Yes	156	52.9
*Alcohol intake* ^*∗∗*^		
Do not drink	114	39.9
Drink eventually	165	57.7
Drink daily	6	2.1
Ex-alcoholic	1	0.3
*BMI (kg/m* ^*2*^)		
Overweight	209	70.8
*Self-report of medical diagnosis of diseases*		
Diabetes	56	19.0
Dyslipidemias	233	79.3
Hypertension	117	39.6
*Use of medicines*		
Oral hypoglycemic or insulin	53	17.9
Statins or fibrates	106	35.9
Antihypertensives	117	39.6

UFV = Universidade Federal de Viçosa; BMI= body mass index. ^*∗*^*n* = 291. ^*∗∗*^*n* = 286.

**Table 2:** Food groups based on dietary patterns of individuals with cardiometabolic risk according to chemical and botanical composition similarities, Brazil, 2017.

Food/group	Foods found within the food record
1. Rice and tubers	White rice, baked or nonfried potatoes (all species of potatoes including *Arracacia xanthorrhiza* and sweet potato), yams, cassava, corn.
2. Beans	Beans (brown bean, red bean, black bean, or white bean), lentil, chickpeas.
3. Vegetable oils	Soy oil, olive oil (virgin or extra-virgin).
4. Leafy vegetable	Watercress, lettuce, green onion, cabbage, spinach, mint, basil, mustard, arugula, parsley.
5. Nonleafy vegetables	Pumpkin, zucchini, leek, eggplant, beet, broccoli, onion, carrot, chayote, cauliflower, eggplant, peppers (green, red, or yellow), palm heart, cucumber, okra, radish, cabbage, tomato, green beans.
6. Whole grain and nuts	Brown rice, oats, flaxseed, sesame seed, quinoa, almonds, peanuts, pistachios, cashews, and other nuts.
7. Fruits and natural juice	**Fruits:** avocado, pineapple, Barbados cherry, plum, prune, banana, khaki, coconut, guava, kiwi, lemon, orange, apple, papaya, mango, passion fruit, watermelon, strawberry, nectarine, pear, peach, tangerine, grape. **Juices:** Barbados cherry fruit, pineapple, guava, passion fruit, and grape plus coconut water.
8. Chicken salad sandwich	Sandwich (bread, salad, and a protein food source that is usually chicken)
9. Milk	Fluid or powdered milk (includes whole milk, half-creamed, or skimmed milk).
10. Dairy	**Cheese**: fresh cheese, half-cured fresh cheese, mozzarela, parmesan, provolone, curd, creamy cheese, ricotta and cottage. **Beverages:** fermented beverage, nonflavoured yogurt (whole or skimmed), fruit yogurt (whole or skimmed), dairy beverage (whole or skimmed), and chocolate milk^∗^.
11. Meat, fish, and eggs (cooked, baked, or grilled)	Grilled, roasted or cooked chicken, beef, or pork (all cuts), canned, cooked, grilled, or baked fish, tofu^*∗∗*^, and boiled or scrambled eggs.
12. Meat, offal, and eggs (fried)	Fried meat: beef, chicken, pork, fish. Offals (heart, gizzard, liver) of all species, regardless cooking technique, and fried eggs.
13. Processed meat	Bacon, hamburger steak, breaded, sausage.
14. Margarine/butter, sauces, mayonnaise	Margarine, butter, cream, pork lard, salad dressing, mayonnaise, processed tomato sauce.
15. Sweets and sugar	Candy, cappuccino, sugar added cocoa powder, chocolate bar (milk or dark), milk fudge, gelatin, jelly, ice cream, condensed milk, honey, peanut butter, popsicle, pudding.
16. Cookies, cakes, and breads	Cornstarch, cereal, cereal flour (Nestlé ™), cassava flour/starch, cornmeal, flour/wheat bran, noodles, cookies, cereal bar, biscuits (milk, cornstarch, flour, Sandwich cookie, waffer, or cream cracker), donuts, toasts, muffins, breads (white bread, loaf, hot dog bread, roll bread), bagel, Brazilian cheese bread, cakes and scones, granola (a mix made of whole grains, nuts, and dried fruit).
*17. Fast Food* e pastry	Fried or baked pastry (kibbeh, “Coxinha” (chopped fried chicken with dough), pie, sfiha, other salty snacks), pizza, hamburger
18. Soft beverages and artificial juices	Soft drinks, diet soft drinks, powdered juice, boxed juice, canned juice.
19. Alcoholic beverages	Beer, wine, “cachaça” (sugarcane hard liquor), vodka, rum.
20. Coffee and tea	Regular coffee, tea (mate or herbal).

^*∗*^Consumed by only three participants. ^*∗∗*^Curd made from coagulated and pressed soy milk, consumed by only one participant.

**Table 3:** Dietary patterns and factorial score of food groups consumed by cardiometabolic risk subjects (*n* = 295) in Brazil, 2017.

Food groups	Dietary patterns					
	Traditional	Caloric	Pastry	Healthy	Healthy snacks	*h* ^2^
Rice and tubers	**0.666**	0.106	−0.013	−0.121	0.001	0.514
Beans	**0.703**	0.006	−0.097	0.065	0.048	0.543
Vegetable oils	**0.768**	0.234	0.181	0.066	−0.048	0.698
Leafy vegetable	0.074	−0.124	−0.036	−0.096	**0.623**	0.611
Nonleafy vegetables	**0.410**	−**0.297**	0.044	0.032	0.201	0.384
Whole grain and nuts	−0.005	0.204	0.236	**0.576**	−0.006	0.545
Fruits and natural juice	0.079	−0.107	−0.064	**0.384**	0.293	0.667
Chicken salad Sandwich	−0.155	0.100	0.072	−0.005	**0.594**	0.627
Milk	−0.087	0.202	−**0.619**	**0.258**	−0.102	0.648
Dairy	−0.249	0.022	0.347	**0.459**	−0.208	0.678
Meat, fish, and eggs (cooked, baked, or grilled)	**0.465**	−**0.279**	0.010	0.038	−0.230	0.583
Meat, offal, and eggs (fried)	0.005	**0.476**	0.251	0.070	0.339	0.533
Processed meat	0.048	**0.512**	0.283	−0.199	0.068	0.716
Margarine/butter, sauces, mayonnaise	−0.191	−0.057	0.045	−**0.261**	0.021	0.844
Sweets and sugar	−0.054	**0.611**	−**0.271**	0.032	−0.086	0.681
Cookies, cakes, and breads	−**0.258**	0.034	−0.422	−0.177	−0.212	0.587
*Fast Food* pastry	−0.211	0.173	**0.589**	0.005	−**0.282**	0.646
Soft beverages and artificial juices	0.124	**0.619**	−0.060	0.101	−0.170	0.611
Alcoholic beverages	0.011	0.083	0.182	−**0.416**	0.034	0.436
Coffee and tea	0.070	0.058	0.046	−**0.568**	−0.126	0.799
**Variance explained (%)**	10.9	8.2	7.0	6.9	6.7	—

^*∗*^Extraction method: principal component analysis. Varimax rotation with Kaiser normalization. Bold values indicate factorial score ≥0.25.

**Table 4:** Simple linear regression for association of cardiometabolic risk factors (dependent variables) with dietary patterns (independent variables) in cardiometabolic risk subjects (*n* = 295) in Brazil, 2017.

Dietary patterns	Waist circumference (cm)			BMI (kg/m^2^)			Body fat (%)			Waist-to-hip ratio		
	*β*	CI 95%	*P* ^*∗*^	β	CI 95%	*P* ^*∗*^	β	CI 95%	*P* ^*∗*^	*β*	CI 95%	*P* ^*∗*^
Traditional	3.159	1.523; 4.795	**<0.001**	0.667	0.040; 1.294	**0.037**	−1.208	−2.209;−0.207	**0.018**	0.029	0.018; 0.039	**<0.001**
Caloric	0.072	−1.602; 1.746	0.933	−0.074	−0.706; 0.557	0.817	−0.170	−1.183; 0.842	0.740	−0.004	−0.015; 0.007	0.467
Pastry	0.686	−0.984; 2.357	0.419	0.137	−0.495; 0.768	0.671	0.753	−0.296; 1.802	0.158	0.004	−0.007; 0.015	0.438
Healthy	−2.709	−4.352;−1.065	**0.001**	−1.065	−1.685;−0.446	**0.001**	−0.974	−2.059; 0.112	0.079	−0.019	−0.030; −0.009	**0.001**
Healthy snacks	0.483	−1.189; 2.155	0.570	0.018	−0.614; 0.649	0.956	0.320	−0.688; 1.329	0.532	0.011	0.008; 0.022	0.050

	SBP (mmHg)			Fasting glucose (mg/dL)			TyG			HDL (mg/dL)		
	β	CI 95%	*P* ^*∗*^	β	CI 95%	*P* ^*∗*^	β	CI 95%	*P* ^*∗*^	β	CI 95%	*P* ^*∗*^

Traditional	0.017	0.001; 0.033	**0.033**	0.047	0.016; 0.079	**0.003**	−0.072	−0.142;−0.001	**0.046**	−0.073	−0.107;−0.039	**<0.001**
Caloric	−0.004	−0.020; 0.011	0.587	−0.043	−0.075;−0.012	**0.007**	0.043	−0.024; 0.111	0.210	0.010	−0.024; 0.045	0.561
Pastry	0.001	−0.016; 0.017	0.941	0.005	−0.028; 0.038	0.769	0.036	−0.034; 0.106	0.315	0.010	−0.025; 0.045	0.569
Healthy	−0.030	−0.045; −0.014	**<0.001**	−0.051	−0.083;−0.019	**0.002**	−0.001	0.970;−0.072	0.970	0.058	0.023; 0.092	**0.001**
Healthy snacks	0.014	−0.001; 0.030	0.071	0.019	−0.013; 0.050	0.245	0.028	−0.040; 0.096	0.420	−0.011	−0.045; 0.024	0.547

	**LDL (mg/dL**)			**CT/HDL ratio**			**LDL/HDL ratio**			**TG/HDL ratio**		
	β	CI 95%	*P* ^*∗*^	β	CI 95%	*P* ^*∗*^	β	CI 95%	*P* ^*∗*^	β	CI 95%	*P* ^*∗*^

Traditional	−0.074	−0.115;−0.032	**0.001**	0.016	−0.021; 0.053	0.385	−0.004	−0.057; 0.048	0.869	0.050	−0.036; 0.135	0.253
Caloric	0.012	−0.028; 0.052	0.566	0.001	−0.035; 0.037	0.958	−0.006	−0.055; 0.043	0.805	0.015	−0.066; 0.097	0.712
Pastry	0.019	−0.021; 0.059	0.356	0.008	−0.028; 0.044	0.659	0.002	−0.048; 0.051	0.949	0.011	−0.073; 0.095	0.795
Healthy	−0.019	−0.060; 0.023	0.374	−0.066	−0.102;−0.031	**<0.001**	−0.071	−0.121;−0.021	**0.005**	−0.127	−0.210;−0.044	**0.003**
Healthy snacks	−0.019	−0.058; 0.021	0.349	0.004	−0.032; 0.040	0.834	−0.012	−0.061; 0.036	0.622	0.060	−0.023; 0.143	0.154

*β* = standardized beta coefficient; IC = confidence interval; BMI = body mass index; SBP = systolic blood pressure; TyG = triglyceride-glucose index; HDL = high-density lipoprotein; LDL = low-density lipoprotein; TG = triglycerides. Values in bold indicate statistical significance (*P* < 0.05).

**Table 5:** Poisson regression for association of cardiometabolic risk factors (dependent variables) with positive factor score^∗^ of dietary patterns (independent variables) in cardiometabolic risk subjects (*n* = 295), Brazil, 2017.

Cardiometabolic risk factors	Traditional		Healthy	
	Adjusted model^1^		Adjusted model^1^	
	PR (CI 95%)	*P*	PR (CI 95%)	*P*
Overweight	0.85 (0.74–0.99)	**0.043**	0.88 (0.68–1.14)	0.363
Abdominal obesity	0.19 (0.03–0.96)	**0.045**	0.13 (0.02–0.76)	**0.024**
High WHR	0.05 (0.01–0.19)	**<0.001**	0.03 (0.00–0.22)	**<0.001**
High WHtR	0.20 (0.04–0.99)	**0.045**	0.14 (0.02–0.81)	**0.028**
Excessive body fat	0.68 (0.34–1.35)	0.274	0.71 (0.37–1.35)	0.304
High LDL/HDL ratio	0.86 (0.75–0.99)	**0.041**	1.46 (0.61–3.48)	0.387
High Uric acid	0.97 (0.57–1.63)	0.923	1.07 (0.68–1.69)	0.756
High CRP	1.16 (0.88–1.52)	0.278	0.91 (0.65–1.26)	0.595
Dyslipidemias	0.06 (0.02–0.51)	**0.009**	0.03 (0.01–0.27)	**0.001**
Diabetes	0.05 (0.01–0.45)	**0.007**	0.02 (0.01–0.18)	**<0.001**
Hypertension	0.06 (0.02–0.50)	**0.009**	0.02 (0.01–0.21)	**0.001**

^∗^Positive factor score = higher adherence to the dietary pattern. ^1^ Model adjusted for age, education, physical activity, and alcoholism. PR = prevalence ratio; 95% CI = confidence interval 95%.

## Data Availability

The PROCARDIO-UFV data used to support the findings of this study are restricted by the registration number 066/2012/CEPH in order to protect patient privacy. Data are available from the corresponding author upon request for researchers who meet the criteria for access to confidential data.
